# Outcomes Following Paediatric Tonsillectomy From a UK Tertiary Centre: Imperial College Healthcare NHS Trust’s Experience During the COVID-19 Pandemic

**DOI:** 10.7759/cureus.68732

**Published:** 2024-09-05

**Authors:** Gani N Nuredini, Paula Coyle

**Affiliations:** 1 Otolaryngology - Head and Neck Surgery, Imperial College London, London, GBR

**Keywords:** clinical practice guideline, covid-19, day-case surgery, obstructive sleep apnoea, tonsillectomy

## Abstract

Background

Paediatric tonsillectomy ranks among the most frequently performed surgical procedures globally. The substantial volume of these operations underscores their considerable impact on healthcare systems and resource allocation. Recent guidelines in the United Kingdom have emphasized the safety and feasibility of performing tonsillectomies as day-case surgeries. The interplay of medical necessity, high incidence, and evolving guidelines underscores the importance of continually evaluating and optimizing tonsillectomy practices. This study assesses the outcomes of paediatric tonsillectomies at a UK tertiary centre during the COVID-19 pandemic and the implementation of day-case guidelines.

Methodology

A retrospective analysis was conducted on patients under 18 years of age who underwent tonsillectomy between April 2021 and September 2022. Data on postoperative events until discharge and re-attendance within 14 days were recorded. High-risk subgroups were analysed: subgroup A (two years of age and weighing 12-15 kg), and subgroup B (severe obstructive sleep apnoea (OSA) on polysomnography defined as an apnoea/hypopnoea index >30 events per hour). Binary logistic regression assessed whether age, weight, sex, or procedure time predicted extended hospital stay (more than one night) or the need for oxygen. Day-case tonsillectomy guidelines were created after multi-disciplinary team approval.

Results

A total of 117 patients underwent tonsillectomy, with a median age of four (n = 72 male). OSA/sleep-disordered breathing accounted for 88% (n = 103), and 68% (n = 70) underwent a preoperative sleep study. Same-day discharge rate was 26% (n = 31). Postoperatively, 86 patients were admitted; 44 required overnight oxygen saturation monitoring, 35 for weight extremes, and seven for poor oral intake. Of those admitted, 70 (81%) patients remained well overnight, and 76 (88%) patients were discharged the next day. In subgroup A (n = 17), the average weight was 13.4 kg; two had transient desaturations. Fourteen were discharged the next day. In subgroup B (n = 34), four had transient desaturations with a further two requiring oxygen. Weight (p = 0.071) within the ‘extended hospital stay model’ and procedure time (p = 0.052) within the ‘need for oxygen’ model approached significance for predicting outcomes.

Conclusions

This study offers early insights into paediatric tonsillectomy outcomes during the COVID-19 pandemic at a tertiary centre. Although the same-day discharge rate was lower than the national average, most patients, including high-risk groups, remained clinically stable and were discharged within 24 hours.

## Introduction

Paediatric tonsillectomy is one of the most commonly performed surgical procedures worldwide. Within the United Kingdom alone, approximately 37,000 tonsillectomies are performed annually [1]. The high volume underscores the significance of this procedure, both in terms of impact on healthcare and resource allocation. For example, in the 2016/2017 fiscal year, the cost per paediatric tonsillectomy was £1,000 to the National Health Service (NHS) [2]. The primary indications for undergoing tonsillectomy fall broadly under two categories, recurrent acute tonsillitis/peritonsillar abscesses or sleep-disordered breathing (SDB). There is a much smaller third group where tonsillectomies are performed for diagnostic purposes [3].

SDB is an umbrella term used to describe a spectrum of sleep-related breathing disorders that includes snoring, central apnoea, hypoventilation, and obstructive hypoventilation. The most severe manifestation of obstructive hypoventilation is obstructive sleep apnoea (OSA), which is diagnosed on overnight polysomnography. OSA is characterised by repeated episodes of complete or partial upper airway obstruction, resulting in abnormal ventilation and patterns of sleep. The breathing events that occur during OSA are categorised into apnoeas (pause in breathing lasting at least 10 seconds), hypoapnoeas (a reduction in airflow lasting at least 10 seconds), and respiratory effort-related arousal (an increased respiratory effort that culminates in arousal but does not meet the definition of apnoea or hypopnoea) [4]. SDB is seen as the sequelae of adenotonsillar hypertrophy and, if left untreated, can result in disrupted sleep patterns, daytime somnolence, behavioural issues, and, in severe cases, cardiorespiratory complications such as autonomic dysfunction (hypertension and heart rate variability), endothelial dysfunction, and ventricular remodelling. There is also evidence that shows OSA is linked with childhood obesity and metabolic syndrome [5].

Adenotonsillectomy is an established and safe method for the treatment of OSA. Traditionally, children undergoing adenotonsillectomy were admitted due to the potential risk of a primary bleed and to monitor oral intake and optimise analgesia. There were also concerns for respiratory complications, such as needing supplemental oxygen following surgery and in certain cases needing to commence continuous positive airway pressure (CPAP) ventilation where the indication for surgery was OSA [6].

Over the last decade, data have demonstrated the safety of day-case surgery in selected children both at district general hospitals and tertiary paediatric centres. In 2019, the Getting It Right First Time (GIRFT) report proposed a target of 70% for specialist trusts [7]. This provides an objective and measurable goal for quality improvement. The combination of medical necessity and evolving guidelines highlights the importance of continuously evaluating and optimising tonsillectomy practices.

Objectives

The primary objective of this study is to evaluate the performance of our centre against national targets for day-case tonsillectomy rates during the COVID-19 pandemic. The secondary objectives are (a) to assess postoperative outcomes in high-risk paediatric patients, specifically those aged two years and weighing between 12-15 kg. (b) To evaluate the outcomes of patients with severe OSA as identified through preoperative polysomnography. (c) To develop and implement a day-case tonsillectomy guideline tailored for use in our specialist centre and surrounding district general hospital.

## Materials and methods

A retrospective analysis was conducted of patients who had undergone tonsillectomy between April 2021 and September 2022 and were under the age of 18 at the time of surgery. Patients were also included in our analyses if they had undergone other surgical procedures concurrently, provided that the additional procedure would not have otherwise impacted the length of hospital stay, for example, adenoidectomy or grommet insertion. Patients with complex medical comorbidities were not excluded. These patients are factored into the national day-case rate target of 75%. Patients were operated at two hospital sites (St. Mary’s Hospital, Chelsea and Westminster Hospital). Bipolar, cold steel, or a hybrid technique (bipolar and cold steel) was used for all cases (based on surgeon preference at the time of surgery). Data were collected from electronic patient records (Cerner, Oracle) entered by all clinical staff involved with patient care. Reasons for postoperative admission and clinical events until discharge were noted. Postoperative complications, including re-admission rate and re-attendance to the hospital within 14 days of the procedure were recorded.

**Figure 1:**
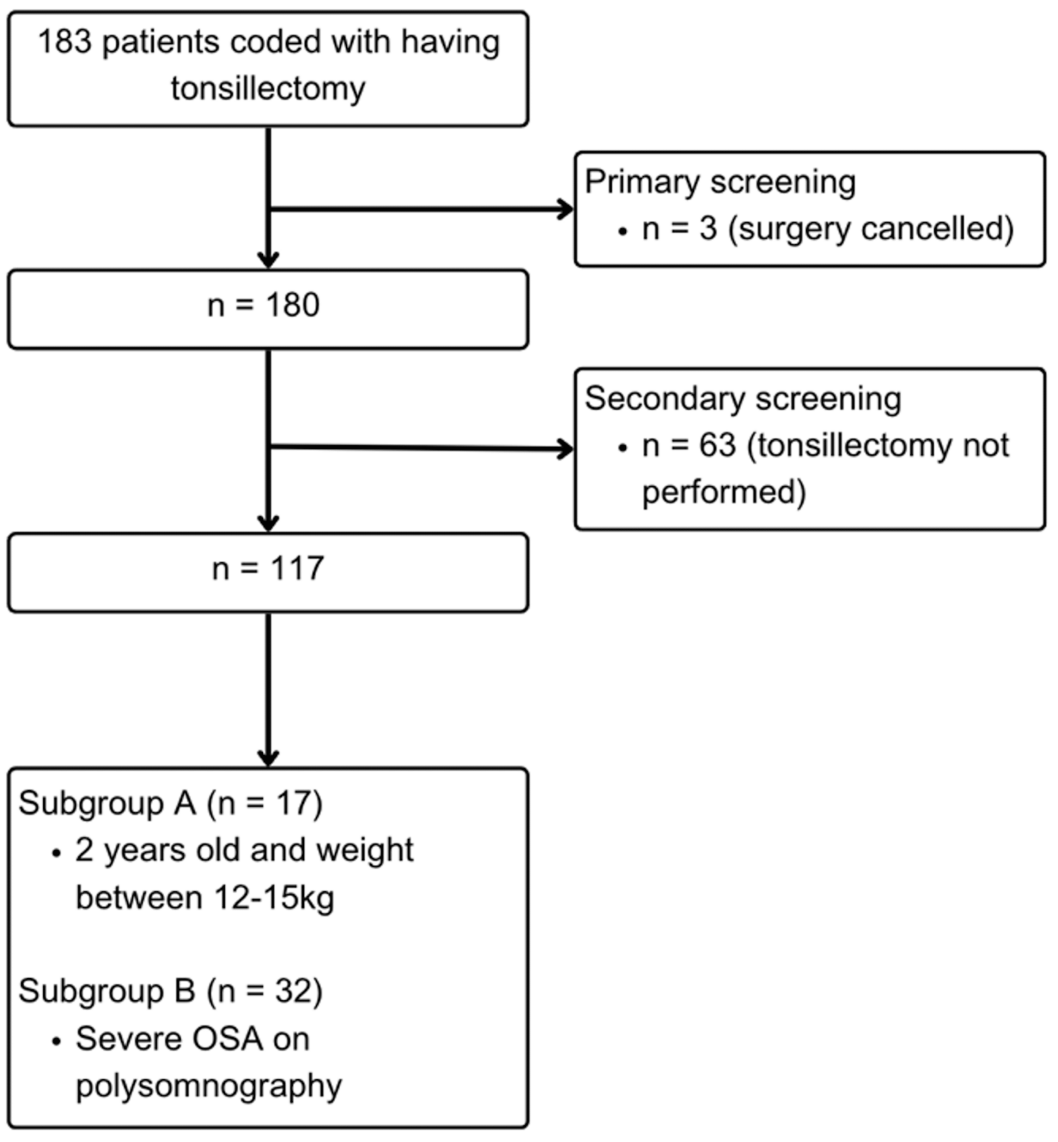
Flow diagram for case selection. OSA = obstructive sleep apnoea

Two separate subgroup analyses were conducted of high-risk patients. Subgroup A included patients aged two years at the time of surgery and weighing 12-15 kg. Subgroup B included patients with severe OSA on preoperative polysomnography (Figure 1). Current national guidelines were used to create ‘Day-case tonsillectomy’ guidelines which were discussed within the local otolaryngology faculty group, followed by multi-disciplinary team meetings involving paediatricians, anaesthetists, and pharmacists, before being presented and approved at the trust-wide paediatric guideline group. UK-WHO age-specific growth charts were used to determine if the patient was above the 91st centile for weight [8]. Binary logistic regressions were performed using JASP Software (Amsterdam, The Netherlands). Variables were selected based on known risk factors for postoperative complications. Statistical significance was accepted at p < 0.05 (with a power of 0.8). SQUIRE 2.0 reporting guidelines were used for reporting [9]. This study was registered locally within the trust’s audit department and did not require ethical approval due to the retrospective design.

## Results

A total of 180 patients were initially listed for tonsillectomy. In total, 66 patients were excluded as tonsillectomy was not performed. A total of 117 patients were included in the final analyses. Median age was four (range = 2-15 years old) with 62% male (n = 72). Overall, 46 patients had associated comorbidities, with asthma (n = 12), prematurity (n = 7), autistic spectrum disorder (n = 6), and trisomy 21 (n = 4) being the most prevalent. In total, 90 patients underwent tonsillectomy for OSA/SDB, 13 for a combination of OSA/SDB and recurrent tonsillitis, 13 for recurrent tonsillitis in the absence of OSA/SDB, and one patient underwent tonsillectomy to obtain histology. Of those in the OSA/SDB subgroup, 68% (n = 70) had a preoperative sleep study. Same-day discharge rate 26%. In total, 86 patients were admitted postoperatively. Forty-four required overnight oxygen saturation monitoring, 35 for extremes of weight (<12 kg and two years old, <15 kg and three years old or >91st centile), and seven for poor oral intake postoperatively. Of those admitted overnight, 81% (n = 70) remained clinically well and did not require medical input. Overall, 88% (n = 76) were discharged the following day. Eighteen patients returned to ED within 14 days of discharge: eight for pain, five due to poor oral intake, three for pyrexia, and two for post-tonsillectomy bleeding. Both patients who returned with post-tonsillectomy bleeding were managed conservatively and discharged the following day (Table 1).

**Table 1:** Outcomes following tonsillectomy. OSA = obstructive sleep apnoea; SDB = sleep-disordered breathing

Demographics	Total (n)	Subgroup A	Subgroup B
	117	17	34
Sex (male)	72	11	23
Weight (mean, kg)	23.4	13.4	22.9
Age, median (range)	4 (2–15)	2 (2–2)	4 (2–13)
Age (years)			
2	25	17	8
3	23	-	6
4	22	-	8
5-9	34	-	7
10–14	12	-	5
14–18	1	-	-
Indication for surgery			
OSA/SDB	90	14	34
OSA/SDB and recurrent tonsillitis	13	2	-
Recurrent tonsillitis	13	1	-
Histology	1	0	-
Polysomnography (n)	70	9	34
Normal	1	-	-
Hypoventilation	1	-	-
Mild	14	1	-
Moderate	20	3	-
Severe	34	5	34
Average operative time (minutes)	39	36	42
Admitted overnight (n)	86	15	34
Weight <12 kg	5	-	3
Weight <15 kg	28	15	11
Oxygen monitoring	44	-	18
Weight >91^st^ centile	2	-	2
Poor oral intake	7	-	-
Events overnight			
None	70	13	27
Desaturation (transient)	6	2	4
Desaturation (requiring supplementary oxygen)	3	-	2
Medical review	2	-	-
Observed bleeding	2	-	1
Same-day discharge (n)	31	2	0
Discharged the next day (n)	76	14	31
Stayed >1 night (n)	10	1	3
Average length of stay (days)	1.2	1.1	1.1
Return within 14 days	18	2	2
Pain	13	2	2
Fever	3	-	1
Post tonsillectomy bleed	2	-	-

Subgroup A

Of the 117 patients included in the analysis, 17 met the criteria. In total, 11 patients were male. The mean weight was 13.4 kg. Overall, 16 patients had no comorbidities. One patient had a genetic disease (*CARD14* mutation). OSA/SDB and a combination of OSA/SDB and recurrent tonsillitis accounted for 95% (n = 16) of cases, of which 56% (n = 9) had preoperative polysomnography. Five patients had severe, three had moderate, and one had mild OSA. Of the 15 patients who were admitted, only one occupied a high-dependency bed and remained well with no oxygen requirement throughout the inpatient stay. Two patients had a single transient oxygen desaturation that recovered spontaneously. These occurred at two hours and 12-18 hours after surgery. Thirteen remained clinically well overnight. Fourteen were discharged the following day, with 11 being discharged within 24 hours.

Subgroup B

A total of 34 patients met the inclusion criteria. The median age was four years old (68% male). Overall, 94% (n = 32) had concurrent adenoidectomy. All patients were admitted postoperatively: 18 for overnight oxygen saturation monitoring and 16 for extremes of weight (<12 kg and two years old, <15 kg and three years old or >91st centile). Overall, 79% (n = 27) remained clinically well and did not require medical input overnight. Four had a single transient oxygen desaturation that recovered spontaneously, occurring at 6-12 hours (n = 1), 12-18 hours (n = 2), and >24 hours (n = 1) after surgery. Two had desaturations that required oxygen supplementation (both within four hours after surgery); these patients had associated comorbidities, asthma and trisomy 21, respectively. Both were off oxygen within 12 hours. One primary bleed occurred that was managed conservatively. In total, 31 (91%) patients were discharged the next day.

Binary logistic regression

Logistic regression analysis was conducted to ascertain if age, weight, sex, or procedure time could be predictors for the need for supplementary oxygen or length of stay (more than one night). The model predicting desaturation requiring oxygen did not achieve statistical significance (Nagelkerke R^2^ = 0.292, p = 0.114), whereas the model predicting a length of stay did (Nagelkerke R^2^ = 0.261, p = 0.007). Within both models, none of the individual variables reached significance levels. Weight in the first model (p = 0.071) and total operation time (p = 0.052) in the second model approached significance (Tables 2, 3).

**Table 2:** Binary logistic regression for oxygen desaturation requiring supplementary oxygen conducted on all patients (n = 117).

Variables	Odds ratio	95% confidence interval	P-value
Age	2.84	-0.51–2.60	0.18
Weight	0.47	-1.58–0.07	0.07
Operative time	0.97	-0.14–0.07	0.49
Sex (male)	1.91	-2.26–3.56	0.67

**Table 3:** Binary logistic regression for length of stay longer than one night conducted on all patients (n = 117).

Variables	Odds ratio	95% confidence interval	P-value
Age	1.51	-0.32–1.15	0.27
Weight	0.81	-0.47–0.04	0.10
Operative time	1.04	0.00–0.07	0.05
Sex (male)	1.94	-1.05–2.38	0.45

## Discussion

Our study evaluated the outcomes of 117 paediatric patients who underwent tonsillectomy, either as a standalone procedure or in conjunction with other surgeries. The overall same-day discharge rate observed was 26%. When excluding patients from subgroup A and subgroup B, the same-day discharge rate increased to 40%. The majority of patients who required overnight admission remained stable and were discharged the following day.

The current national average for same-day discharges following tonsillectomy in specialist trusts is 55% [7]. Our average of 26% is significantly lower. However, it is important to note that national averages were obtained during the pre-pandemic era (2014-2019), whereas our study was conducted during the COVID-19 pandemic. Currently, there is no published data for specialist trusts during the pandemic period to allow for direct comparison. Factors such as reduced staffing could have impacted the standard postoperative pathways that facilitate timely discharges. For instance, the sickness rate among acute staff during the data collection period was 4.6%, peaking at 6.5% in January 2022 (Imperial College NHS Trust) [10]. Similarly, middle-grade doctors were redeployed away from elective specialty work to intensive care or other acute medical settings [11]. These doctors are often responsible for preparing paperwork to facilitate timely discharges. Another factor potentially contributing to our low same-day discharge rates is the surgical technique. Our centre uses bipolar and cold steel techniques, which have been associated with greater postoperative pain. Consequently, seven patients were admitted postoperatively due to poor oral intake secondary to pain. Data from other specialist trusts that exclusively use the Coblation technique, associated with lower postoperative pain, have reported same-day discharge rates of 44% [12-14].

In total, 18 patients returned within 14 days post-surgery, with the majority (n = 13) presenting due to postoperative pain. This is higher (11% vs. 0.3%) than the rates reported at other specialist trusts with a day-case rate of <50% [7]. On further review, the discharging analgesic regimen varied. For example, some had analgesia prescribed ‘as required’ versus ‘regularly’. In other instances, analgesia was prescribed by age versus weight. This highlights the need for effective strategies such as a standardised regimen. A previous study has demonstrated the benefit of implementing a standardised weight-based analgesic regimen in reducing emergency department attendances due to postoperative pain [15] National guidelines now advocate for the regular administration of weight-based paracetamol and non-steroidal anti-inflammatory drugs, with the addition of morphine for breakthrough pain [16]. This approach was extensively discussed during our trust’s guidelines meeting. We concluded to use age-based dosing for paracetamol and ibuprofen. This decision was influenced by the recommended dosing of 15-20 mg/kg QDS for paracetamol potentially exceeding the Medicines and Healthcare products Regulatory Agency’s maximum advised dose of 75 mg/kg/day. Additionally, it was felt that individual weight-based prescriptions could delay discharges due to the extra time required to prepare these medication packs. A similar discussion was held regarding the routine inclusion of morphine. It was agreed that morphine would be prescribed postoperatively in selected cases, such as difficult surgeries or in patients with allergies. Only two (1.7%) patients presented with post-tonsillectomy haemorrhage. Both cases were managed conservatively and were discharged after 24 hours of observation. This is lower (6%) than the rates reported at other specialist trusts with a day-case rate of <50% [7].

Weight under 15 kg was previously associated with an increased risk of postoperative complications. The ENT UK consensus statement in 2009 advised that children under the age of two or three and weighing less than 15 kg should be referred onwards for consideration of tonsillectomy at regional centres [17,18]. However, it was also acknowledged that a lack of data exists for children aged two and weighing 12-15 kg. Within our cohort (subgroup A), 15 of the 17 patients were admitted for overnight observation. Transient oxygen desaturation was observed in two patients, which spontaneously resolved without necessitating intervention. One patient with underlying comorbidities (chronic lung disease of prematurity) was admitted to the paediatric high-dependency unit for precautionary measures, remained stable overnight, and did not require medical intervention. Current guidelines propose that specialised centres may consider performing day-case surgeries on children aged two years and weighing above 12 kg. Our findings provide additional support for this recommendation. Two patients in subgroup A presented within 14 days of discharge with postoperative pain, which was managed by optimising analgesia without requiring hospital admission.

Similarly, it was also thought that children undergoing adenotonsillectomy for severe OSA had an increased risk of postoperative complications. However, the incidence of major respiratory complications was no greater in children undergoing tonsillectomy for OSA compared to those having adenotonsillectomy for non-OSA indications [19]. In our cohort (subgroup B), all 34 patients were admitted postoperatively, with 79% remaining clinically stable without requiring medical intervention. Transient oxygen desaturations were observed in four patients, which resolved spontaneously. Notably, only two patients required supplemental oxygen, both within four hours of surgery and both presenting with significant comorbidities (severe asthma with multiple hospital admissions for exacerbations and trisomy 21). The high rate of next-day discharge (91%) further substantiates the potential for day-case tonsillectomies in carefully selected patients with severe OSA. Nevertheless, individualized risk assessment remains crucial, considering factors such as comorbidities.

The binary logistic model predicting desaturation requiring oxygen did not achieve statistical significance (p = 0.114), suggesting that the selected variables (age, weight, total operative time, and sex) were not predictors of this desaturation within our cohort. Despite the lack of overall model significance, weight approached significance (p = 0.071) with an odds ratio of 0.468. This trend, while not statistically significant, aligns with clinical observations that children with lower weight are at an increased risk of postoperative respiratory complications [20]. Conversely, the model predicting prolonged hospital stay (>one night) reached statistical significance (p = 0.007), although individual predictors did not achieve significance. Total operative time approached significance (p = 0.052) with an odds ratio of 1.039, suggesting that longer surgeries may increase the likelihood of extended hospitalisation. While our models provide some insights, they also need to be considered within the context of our sample size.

Limitations

Referrals to our tertiary centre are received from West London and surrounding areas, as well as across South West England. Our electronic health records are integrated across seven hospitals in West London, three of which provide paediatric emergency services. Patients presenting with postoperative complications outside our catchment area are advised to attend their nearest emergency department with otolaryngology out-of-hours coverage. Consequently, such data may not have been captured, potentially contributing to a lower post-tonsillectomy haemorrhage rate compared to national reports. Additionally, this study was retrospective in design and involved a small sample size due to the reduced level of operating during the COVID-19 pandemic, which limits the generalisability of the findings. A multicentre study would have been beneficial when looking at the impact COVID-19 was having on day-case tonsillectomies across the United Kingdom. The use of binary logistic regression analysis in this context may not fully capture the variability and complexity of the population, thereby affecting the robustness and applicability of the results to a broader patient cohort.

## Conclusions

The findings of this study provide some of the first insights into the outcomes of paediatric tonsillectomy during the COVID-19 pandemic at a tertiary centre. While the same-day discharge rate was lower than the national average, the majority of patients, including those in high-risk groups, remained clinically stable and were discharged within 24 hours. Future work will focus on a prospective study measuring outcomes following the implementation of our day-case guidelines that standardised analgesic regimens.
